# High sensitivity ultraviolet graphene-metamaterial integrated electro-optic modulator enhanced by superlubricity

**DOI:** 10.1515/nanoph-2022-0185

**Published:** 2022-07-12

**Authors:** Yanli Xu, Chuan Zhang, Weimin Li, Rong Li, Jiangtao Liu, Ze Liu, Zhenhua Wu

**Affiliations:** College of Mechanical and Electrical Engineering, Guizhou Minzu University, Guiyang 550025, China; Key Laboratory of Microelectronic Devices and Integrated Technology, Institute of Microelectronics, Chinese Academy of Sciences, Beijing, 100049, China; The LEADS of Southeast University, The National Mobile Communications Research Laboratory of Southeast University, Nanjing, 211189, China; Department of Engineering Mechanics, School of Civil Engineering, Wuhan University, 430072, Wuhan, China; University of Chinese Academy of Sciences, Beijing, 100029, China

**Keywords:** graphene, nanomechanical system, plasmonic metamaterials, superlubricity, ultraviolet

## Abstract

Ultraviolet (UV) electro-optic modulation system based on graphene-plasmonic metamaterials nanomechanical system (NEMS) with superlubricity is investigated. Due to the strong optical absorption intensity of graphene in the UV region and the combination of metamaterial structure based on surface plasmons, the modulation depth of the UV NEMS electro-optic modulator approaches as high as 8.5 times compared to the counterpart modulator in visible light region. Meanwhile, the superlubricity significantly reduces the power consumption of the UV electro-optic modulation system due to its extremely low friction coefficient. It also significantly increases the response speed of the modulator, with response time down to nanoseconds. The modulation voltage can be equal to or less than 150 mV. The proposed electro-optic modulation system has a simple structure and high sensitivity, which is supposed to have important applications in UV optoelectronic devices and systems.

## 1 Introduction

Ultraviolet (UV) light is the electromagnetic wave whose wavelength is between 10 and 400 nm in the electromagnetic spectrum [1–3]. It has important applications in spectral analysis, UV imaging, UV confidential optical communication, and other military and civil fields [4–6]. Since the working wavelength of photoelectric devices depends on the bandgap of semiconductor materials, UV optoelectronic devices are usually prepared using traditional wide bandgap semiconductors, such as GaN, SiC, ZnO, Ga_2_O_3_, and AlGaN [7–10]. However, UV modulation is mainly realized through internal modulation, with a low modulation speed and high noise. For example, the modulation speed of UV modulation using mercury-xenon lamp as the light source is about 400 kHz [11–13]. Graphene has a very strong light response in the UV region due to the strong electron–hole interaction near the saddle point of graphene, thus graphene is an ideal UV photoelectric material [14–17]. However, graphene in the UV region has a high Fermi energy level. Therefore, it is not easy to adjust the light absorption near the Fermi energy level using traditional modulation of graphene Fermi energy level [18].

Recently, Liu et al. introduced the high-speed spontaneous recovery motion based on superlubricity into the graphene micro–nano mechanical system, and designed a micro–nano mechanical electro-optic modulator with a high speed and low power consumption based on superlubricity [19]. This kind of modulator does not need to adjust the Fermi energy of graphene. Superlubricity is the interface with a very small coefficient of friction [20–30], which will lead to some new applications of micro–nano mechanical systems, such as high-speed self-recovery graphite blocks [31, 32]. The modulator uses the superlubrication between the interfaces to prevent wear, reduce friction energy loss and improve the sensitivity, which leads to the drastically increased working life and reduced power consumption.

Here, an ultraviolet electro-optic modulator for micro–nano mechanical system based on superlubrication is proposed. We found that by combining the superlubricity with plasmonic metamaterials, the performance of the device especially the modulation depth of the electro-optic modulator can be significantly improved. The polarization-dependent characteristics of the modulator based on plasmonic metamaterials make it possible to develop novel polarization-dependent UV devices. The optical modulation system has a simple structure and high sensitivity, which will have important applications in UV communication.

## 2 Theoretical model and analytical method

**Figure 1:**
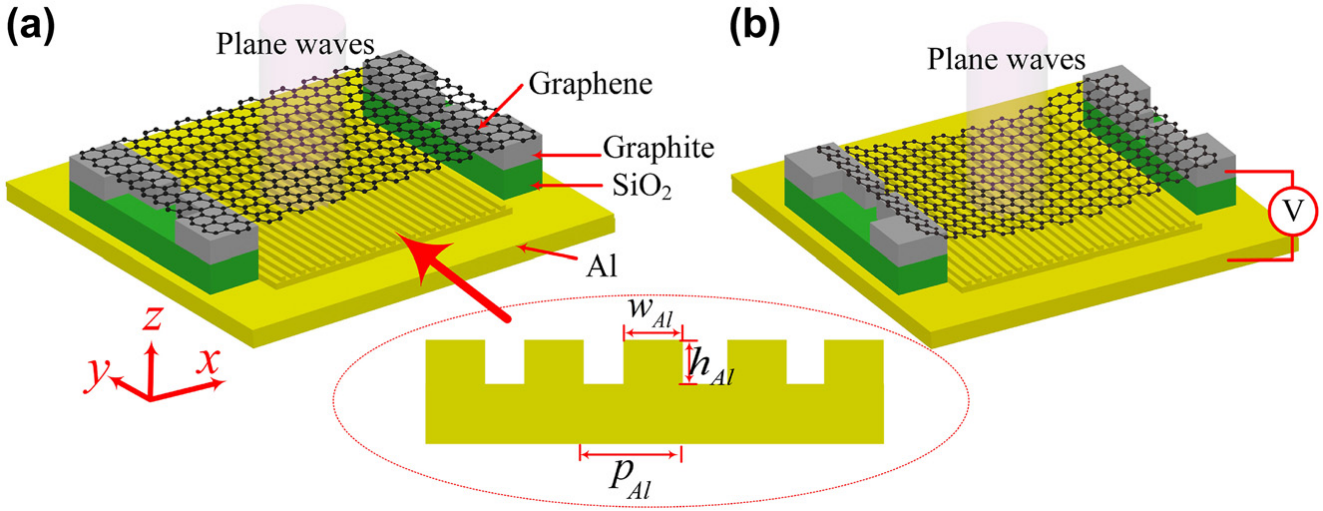
Schematic of UV modulation structure. (a) No voltage applied. (b) Schematic of graphene moving downward and shrinking after applying voltage. Electrodes are connected to a graphite and plasmonic metamaterial substrate.

The structure studied is shown in Figure 1. Due to the high reflectivity of aluminum in the UV region, the conductive reflective substrate consists of aluminum material. The upper aluminum metal surface is designed into a periodic plasmonic metamaterial grating structure, where *h*_Al_ = 30 nm, *w*_Al_ = 60 nm, and *p*_Al_ = 180 nm. The optical constants of Al are obtained from Ref. [33]. The graphene and plasmonic metamaterials are separated by air. The graphene ends are in contact with graphite lubrication layer and separated from the aluminum substrate through an insulating layer, SiO_2_, with the refractive index of 1.48. Due to the existence of the singular saddle point, the graphene suspended in the uppermost layer has an asymmetric light absorption peak near 4.6 eV (270 nm), which is close to 9% [14–17], far higher than the reported absorption of 2.3% [34–36] in the visible and infrared regions, which greatly improves the optical response of graphene in the UV region. Using the imaginary part of the permittivity of graphene at 4.6 eV provided in Ref. [14], the real part of the permittivity can be expressed in the integral form of the imaginary part of the permittivity by the Kramers–Kronig (KK) relation [37],

(1) $$Re\left[\epsilon \left(\omega \right)\right]=1+\frac{1}{\pi }P\overset{\infty }{\underset{0}{\int }}\frac{s\;Im\left[\epsilon \left(\omega \right)\right]}{s^{2}-\omega^{2}}ds,$$

where *P* is the principal value integral. The permittivity of graphene in the UV region can be obtained as a function of frequency. The refractive index of graphene can be calculated by $n\left(\omega \right)=\sqrt{\epsilon \left(\omega \right)}$ , and its thickness is 0.34 nm. Therefore, to better regulate the restoring force, the design of the superlubrication interface by using graphite electric contact terminals as shown in Figure 1(a) is consistent with that in Ref. [19]. The graphite electric contact terminals consist of four L-shaped graphite component. Similar air bridge structures have been successfully realized in previous experiments [38–40]. Thus, this structure is convenient for experimental realization.

When a bias is applied between the graphene and plasmonic metamaterials, the graphene and plasmonic metamaterials attract each other, due to electrostatic force. The surface is approximately parallel to the substrate because graphene bending is very small, and the size of the graphene is much larger than the distance between the graphene and plasmonic metamaterials. Thus, the structure can be approximated as a parallel plate capacitor. Furthermore, when a voltage is applied, the graphene also suffers from a retraction force from the graphite substrates provided by interlayer binding energy and very little friction. In particular, interlayer binding prevents the interface between graphene and graphite dissociated layer from being reduced, thus providing a retractive force. The interlayer binding energy *E*_ib_ is proportional to the contact area *S*_i_ and *E*_ib_ = *γ*_g_ *S*_i_, where *γ*_g_ is the interlayer binding energy coefficient. If the width of the contact surface is *L*_cs_, when a movement of d*x* in the *x* direction occurs, the contact area and contact energy change *L*_cs_d*x* and d*E*_ib_ = *γ*_g_ *L*_cs_d*x*, respectively. The retraction force in the *x* direction is given by the equation [19],

(2) $$F_{r}=\frac{\partial E_{ib}}{\partial x}=\gamma_{g}L_{cs},$$

The equilibrium position of graphene can be fixed and calculated once these forces reach equilibrium. The motion of graphene can be obtained by solving Newton’s equations numerically, as described in Ref. [19]. The interlayer bonding energy of graphene used in the calculation is 0.23 J/m^2^ [32], and the friction coefficient *α*_f0_ is 0.006 MPa·s/m [31].

The finite difference time-domain method is used to simulate the reflection characteristics of the structure. Periodic boundary conditions apply in *x* and *y* directions. Plane waves incident from the *z* direction use the perfect matching layer to absorb all light outside the boundary along the propagation direction. Non-uniform grids are used to balance storage and computing time.

## 3 Results and discussion

**Figure 2:**
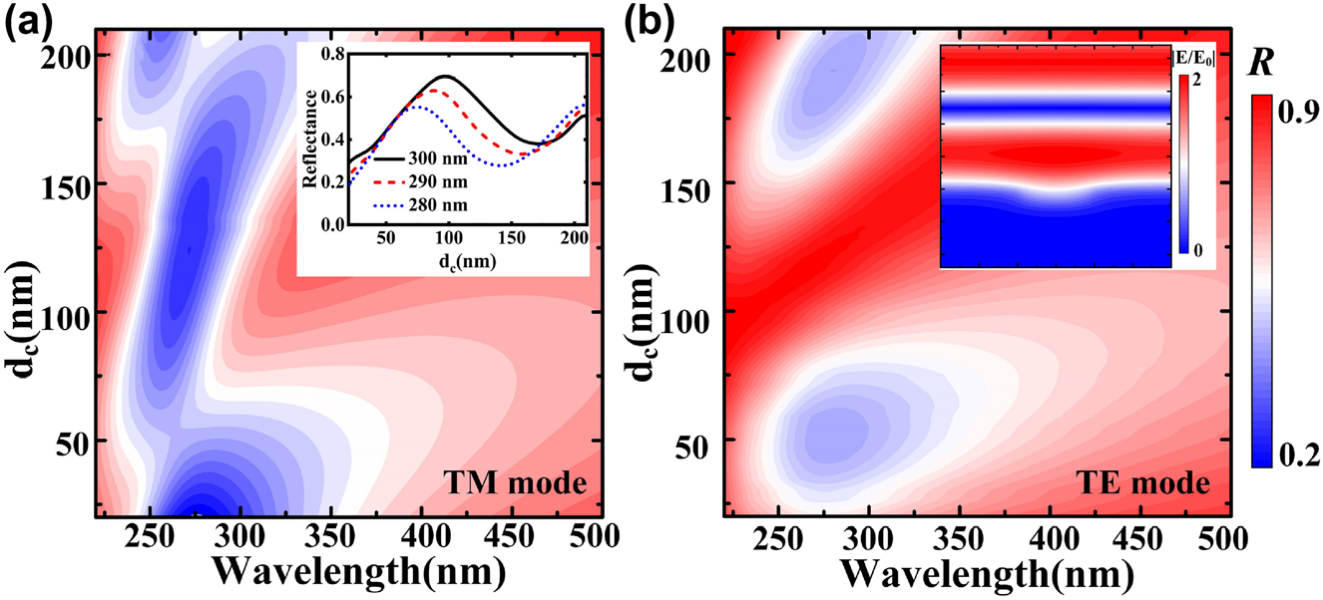
Trend of reflection with the equilibrium position and wavelength of graphene, (a) TM mode, the system reflection changes with distance when the wavelength is 280, 290, and 300 nm, respectively. (b) TE mode, the illustration shows electric field distribution.

The incident light can be divided into TM and TE modes according to their polarization. The TM (TE) mode is the magnetic (electric) component parallel to the metamaterial grating or graphene interface. When the TM-mode UV light incident from above the graphene along the *z* direction, the diffraction phenomenon occurs on the surface of the plasmonic metamaterials, resulting in diffraction light waves. However, when the wave vector of the diffused light wave is consistent with that of the surface plasma wave, the surface plasma wave will be excited at the interface between the surface of the plasmonic metamaterial and the air medium. Surface plasmon resonance will be generated. Under the resonance excitation, the surface plasmons of plasmonic metamaterials can break through the diffraction limit and restrict the incident light to the sub-wavelength scale [41–50]; thereby, forming a strong local field effect. Furthermore, when a voltage is applied between the plasmonic metamaterials and graphene, the graphene and plasmonic metamaterials are attracted to each other by Coulomb forces, which force the graphene downward. Meanwhile, when no bias voltage is applied, graphene is leveled by restoring force. Thus, the graphene and plasmonic metamaterials attract or repel each other under the action of voltage; thereby, causing the graphene to slide on the graphite lubrication layer; the distance between the graphene and plasmonic metamaterials changes. The reflectance of the structure in TM mode varies with the distance between graphene and plasmonic metamaterials (Figure 2(a)). Due to the local surface plasma of the metamaterial in this direction, the graphene moves from 20 to 100 nm away from the metamaterial surface at a wavelength of 300 nm, enabling efficient modulation at 80 nm. This is because of the strong local field effect of light on the surface of the plasmonic metamaterials, which allows the higher modulation depth to be achieved only by moving the graphene a very small distance. However, when TE polarized light is incident, its field intensity direction is parallel to the interface between metamaterial grating and air. Thus, it will not influence the movement of free electrons on its surface. In other words, it will not produce a plasma resonance phenomenon at the interface. Figure 2(b) shows the variation of light reflectance with the distance and wavelength between graphene and plasmonic metamaterials under TE polarization. The illustration shows the electric field distribution, which is a standing wave distribution without local field generation. The findings demonstrates that the proposed design of metamaterial grating makes the structure sensitive to polarization and makes it possible to develop new polarization related devices in UV region.

**Figure 3:**
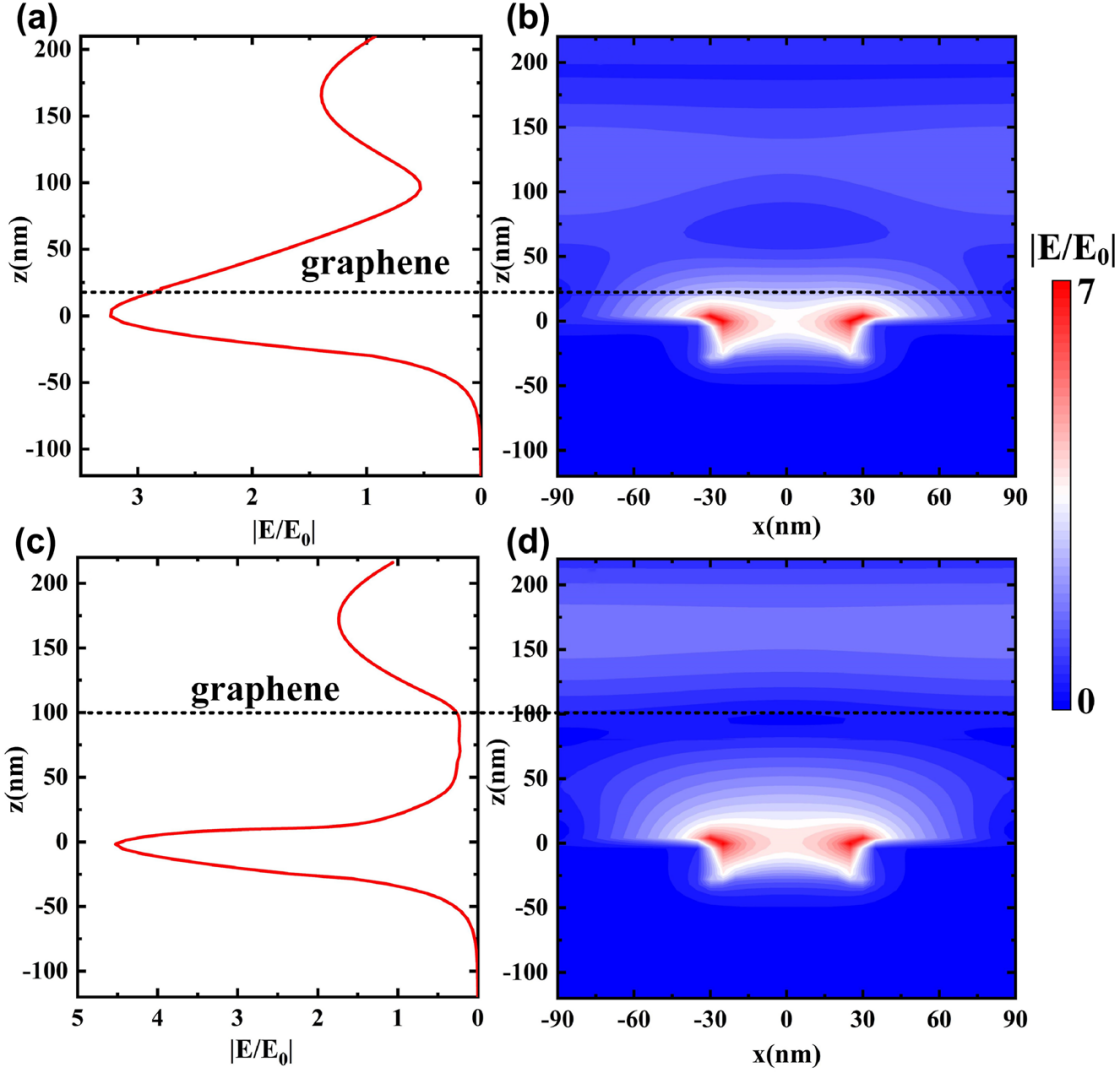
Dependence of the electric field distribution on the space between the graphene and the metamaterial substrate, where *λ* = 300 nm is used. (a) With space of *z* = 20 nm. (b) With space of *z* = 100 nm. The dotted line shows where the graphene is in equilibrium.

Considering that graphene absorption is closely related to the structure electric field distribution, to analyze the physical mechanism behind the modulation depth of plasma resonance enhancement, we calculated the electric field distribution of graphene at the distance of 20 and 100 nm from the plasmonic metamaterials in TM mode. At a wavelength of 300 nm, when the equilibrium position of graphene is 20 nm above the plasmonic metamaterials, Figure 3(a) shows that local surface plasmon resonance forms on the surface of the metal metamaterial structure, which usually occurs at the interface between the metal metamaterial structure and the medium. This strong localization will lead to the electric field enhancement effect and light energy convergence in the near field, which significantly enhances the interaction between light and matter. Moreover, this effect limits the electromagnetic field enhancement to the near field and extends to the nearby air medium, followed by rapid attenuation. Therefore, the gradient field with great difference in electric field distribution is formed. Graphene will show different absorption properties depending on the intensity of the electric field. Figure 3(b) shows that when graphene is located at 20 nm above the plasmonic metamaterials, i.e., when graphene is in a strong electric field, the absorption of graphene is large, resulting in a small reflection of the device. However, when graphene is far away from the plasmonic metamaterials, such as about 100 nm above the plasmonic metamaterials, graphene is in a weak electric field with small absorption, so the light reflectivity of the device is high (Figure 3(c) and (d)). In this way, the distance between the graphene and plasmonic metamaterials can be changed by applying a voltage. The light absorption of the graphene can be adjusted by the electric field distribution gradient between them; thereby, modulating the reflectivity of the modulator and enabling efficient and fast light modulation.

**Figure 4:**
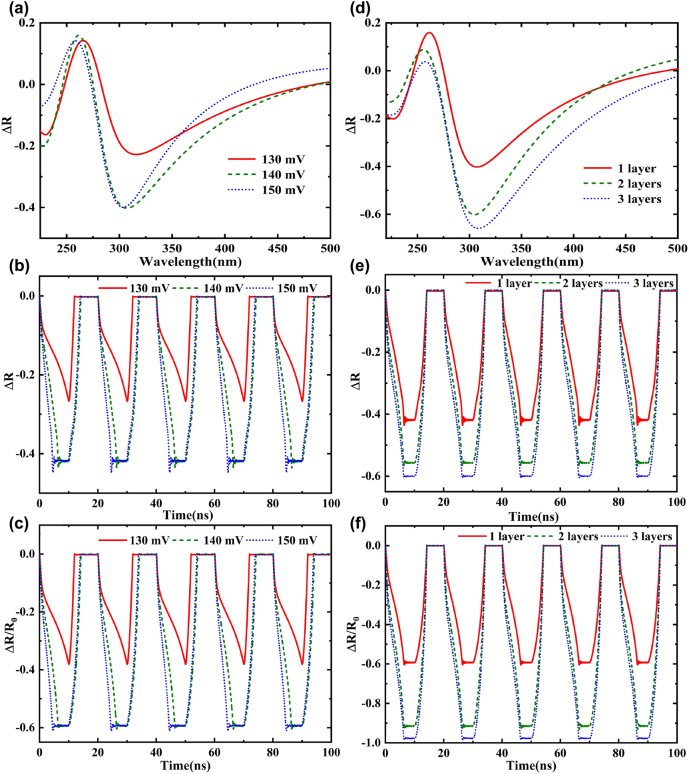
The reflection varies with voltage or the number of graphene layers. (a) Absolute variation of reflection with wavelength at different voltages. (b) The absolute change of reflection under different voltages varies with time. (c) The relative change of reflection with time under different voltages. (d) The absolute variation of reflection with wavelength at different graphene layers. (e) The absolute change in reflection of different graphene layers over time. (f) The relative change of the reflection of different graphene layers over time.

Figure 4(a) shows the absolute change of reflection with wavelength at different voltages. There is a peak near the wavelength of 300 nm, indicating that graphene has a large absorption change at this wavelength due to the strong local field of light formation in the plasmonic metamaterials. As the voltage decreases, the absolute change peak of reflection redshifts, but the modulation depth decreases gradually. Figure 4(b) and (c) shows the absolute and relative change of reflectivity when the driving electric field is a square wave with a period of 20 ns, respectively. Δ*R* = *R* − *R*_0_ is the change of reflectivity, and the relative change of reflectivity is Δ*R*/*R*_0_, where *R*_0_ refers to the reflectivity under zero bias voltage. Here, the initial position of graphene is set to 100 nm away from the plasmonic metamaterials. The response time of the modulator is about 4 ns, which is more than four orders of magnitude higher than the traditional mechanical modulation speed. The absolute change of reflection is more than 0.4, and the relative change of reflection is about 0.6. It is worth pointing out that the relative change of reflectance of the etched microstructures on the substrate is about 18% higher than that of the unetched microstructures on the substrate, which reflects the excellent properties of the structure. Moreover, due to the high absorption of graphene in the UV region [14] and the local field effect of metamaterials, the modulation depth of the UV modulator can be increased by 8.5 times compared with the graphene-based modulator in the visible and infrared regions. Meanwhile, the modulation voltage is only 150 mV, about 1∼3 orders of magnitude smaller than the traditional optical modulation voltage, and the full width at half maximum (FWHM) is reduced to 64 nm. When the voltage changes to 140 mV, the response time is slightly shortened, indicating good voltage adaptability. When the modulation voltage drops to 130 mV, the driving force of the electric field becomes weak; the motion amplitude of graphene becomes small, and the modulation speed and depth decrease. Herein, the in-plane stiffness of graphene is very large, but the out-of-plane stiffness is very low, so the out-of-plane elastic modulus is small, and the elastic deformation potential energy generated is also small. Under the action of superlubrication, the shear force of interlaminar sliding can be ignored, so there is almost no in-plane deformation when the out-of-plane deformation required for light absorption modulation is generated, resulting in a small change in elastic potential energy. Thus, a very small voltage can bend the graphene downward, allowing for a wide range of adjustments.

Since graphene absorption is proportional to the number of graphene layers, multilayer graphene can further improve light modulation depth. For monolayers, the absolute change of reflection is about 0.4, and the relative change is about 0.6; in contrast, for bilayers (three graphene layers), the absolute change is about 0.5 (0.58) and the relative change is about 0.9 (0.98) (Figure 4(d)–(f)). The modulation depth of the device improves as the number of graphene layers increases. The modulation wavelength range of two-layer and three-layer graphene is the same as that of monolayer graphene (Figure 4(d)). As the number of graphene layers increases, the absolute and relative changes and the FWHM of device reflection gradually increase. In addition to increasing the number of graphene layers, the modulation depth can be further improved using graphene and other two-dimensional materials with large absorption, such as heterojunction structures formed with h-BN [51].

**Figure 5:**
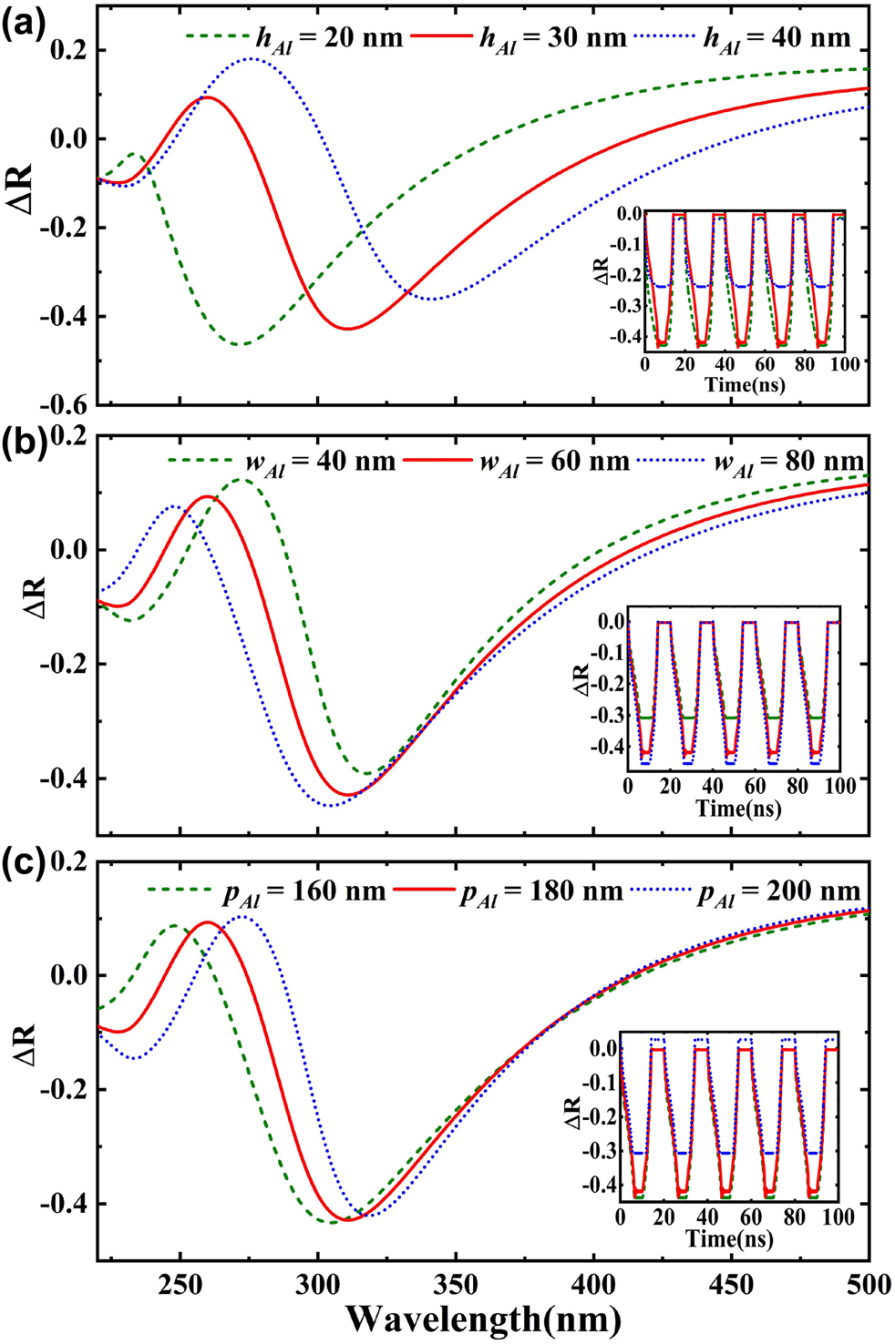
The reflection varies with the geometry of the metamaterial substrate. (a) Absolute change of reflection varies with the height *h*_Al_ of the plasmonic metamaterial grating. (b) The absolute change of reflection varies with the width *w*_Al_ of plasmonic metamaterial grating. (c) The absolute change of reflection varies with the change of period *p*_Al_ of plasmonic metamaterial grating. The illustration shows the absolute change of reflection over time with different parameters at a wavelength of 300 nm.

Furthermore, the influence of the structural parameters of plasmonic metamaterials on the device light modulation was investigated (Figure 5). The absolute change of the device’s reflection varies with the depth of the plasma metamaterial grating (Figure 5(a)). With an increase in *h*_Al_, the formant shifts redshift and changes sharply, confirming that the device is sensitive to the depth of the plasmonic metamaterial grating. It is worth noting that when *h*_Al_ = 20 nm, the modulation wavelength moves to nearly 270 nm, which is of great significance for the modulation of solar blind UV light. Figure 5(b) and (c) shows the influence of absolute change of reflection on the width of plasma metamaterial grating *w*_Al_ and periodic *p*_Al_, respectively. With an increase in *w*_Al_, the peak value shifts blue, the FWHM increases; in contrast, the peak value redshifts and the FWHM decreases as *p*_Al_ increases. Herein, only reflection diffraction waves of TM mode are considered here because surface plasmons can only be excited by TM waves. In general, surface plasmons are excited when the following dispersion relation is satisfied [52],

(3) $$|k_{0}\sin\theta +\frac{2\pi m}{p_{Al}}|=k_{0}\sqrt{\frac{\epsilon_{Al}\epsilon_{0}}{\epsilon_{Al}+\epsilon_{0}}}.$$

where *k*_0_ is the vector of incident wave in vacuum, *θ* is the incident angle, and *m* is an integer of diffraction order. *ϵ*_Al_ and *ϵ*_0_ are dielectric functions of metal and air. It can be seen that the surface plasmon resonance is closely related to the period of metamaterial grating. Since changing the parameters of the plasmonic metamaterial will affect the surface local field and graphene absorption, its modulation amplitude also changes accordingly, as shown in the illustration. Thus, the resonant wavelength and modulation depth can also be tuned by modulating the structural parameters of the plasma metamaterial.

**Figure 6:**
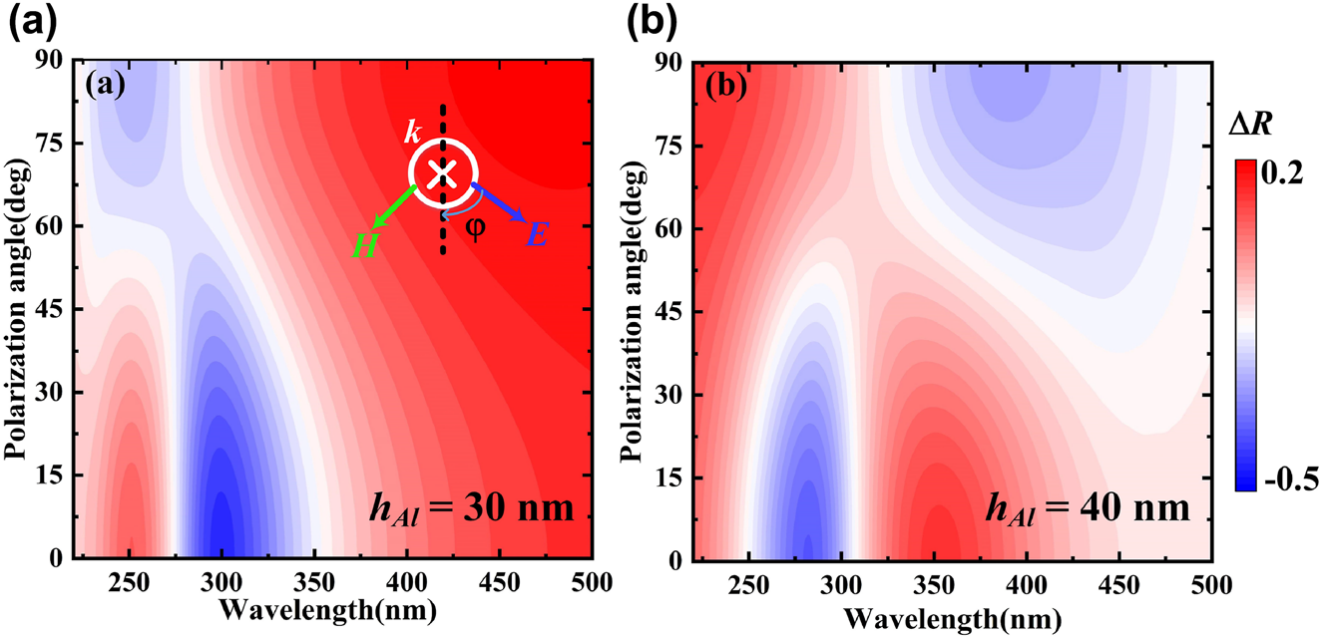
Trend of absolute variation of reflection at different polarization angles. (a) *h*_Al_ = 30 nm, (b) *h*_Al_ = 40 nm.

To further investigate the polarization dependence of the modulator, we plot the spectra of the absolute change of reflection between graphene and plasmonic metamaterials at different polarization angles. As shown in Figure 6, this structure is sensitive to the polarization of incident light. When *h*_Al_ = 30 nm, and the polarization angle (which is the angle between the electric field and the *y*-axis) changes from 0° to 90°, the absolute change of reflection decreases; the bandwidth decreases; the absolute change peak of reflection shifts blue. The absolute reflection changes the most when the wavelength is about 300 nm; its value changes from −0.44 to 0 in TM to TE modes, respectively. However, when *h*_Al_ = 40 nm, the peak of absolute reflection change redshift (Figure 6(b)). A large change in device performance can be observed when *h*_Al_ changes, with the absolute change of reflection varying from −0.22 in TM mode to 0 in TE mode at a wavelength of 300 nm. The wavelength of the maximum absolute change of reflection shifts to 280 nm, with the absolute change of reflection varying from −0.35 in TM mode to 0 in TE mode. According to the above analysis, the structure is more sensitive to *h*_Al_ changes, which is attributed to the formation of the micro-cavity effect between plasmonic metamaterial and graphene. The device can also be tuned reflectively by polarization angle, which gives the structure flexible polarization tunability.

**Figure 7:**
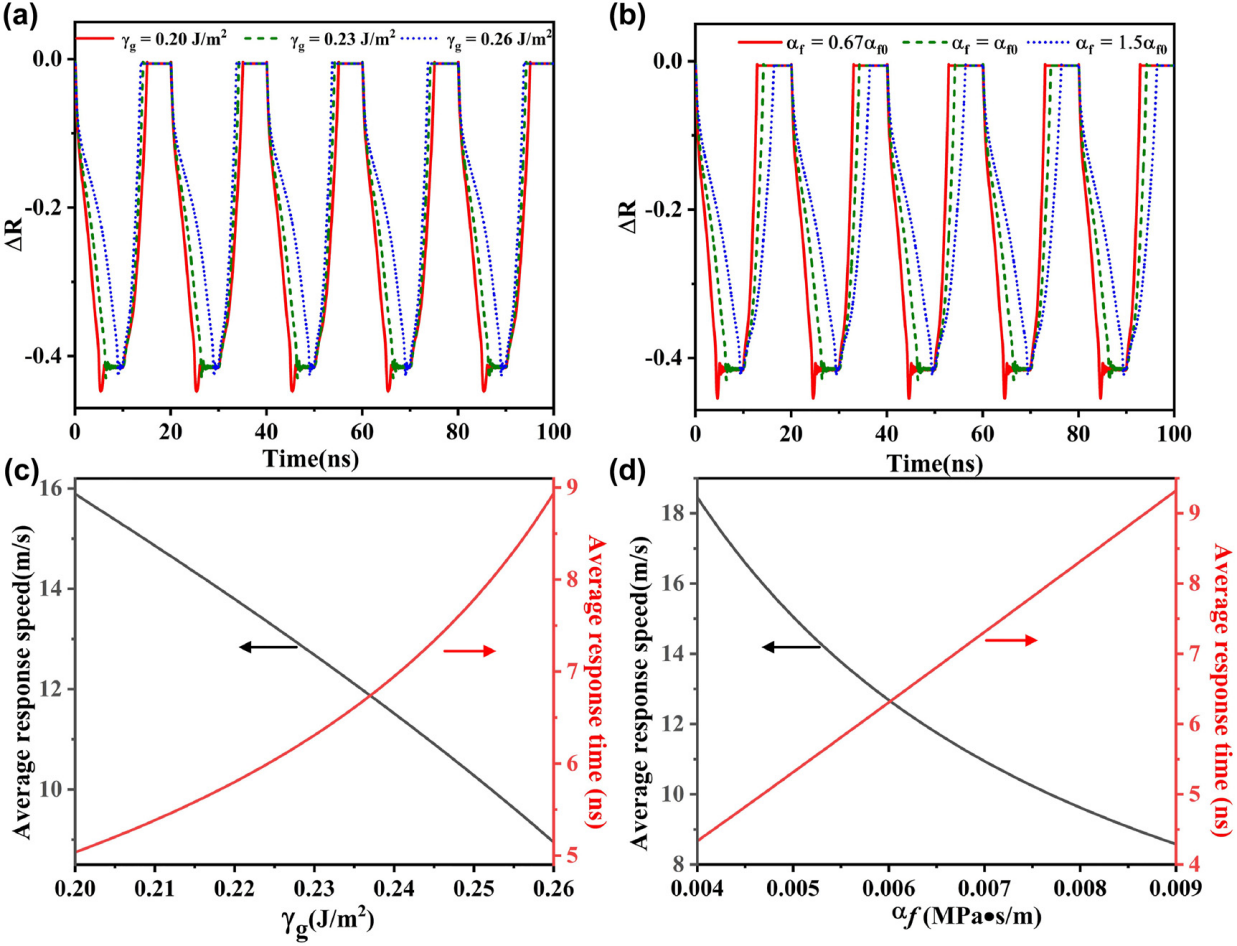
The influence of different interlayer binding energy coefficient and friction coefficient on the system. (a) The absolute change in reflectance as a function of time for different interlayer binding energy coefficients *γ*_g_. (b) The absolute change in reflectance as a function of time for different friction coefficients *α*_f_. (c) The average response speed in *z* direction and average response time as a function of *γ*_g_. (d) The average response speed in *z* direction and average response time as a function of *α*_f_.

Under different processing conditions, temperature, working material, stress and/or other external conditions, the interlayer bonding energy of graphene and friction coefficient will be different. When the interlayer binding energy coefficient varies from 0.20 to 0.26 J/m^2^, the absolute change of the device’s reflection only slightly changes [Figure 7(a)]. When the interlayer binding energy coefficient *γ*_g_ is small, the retraction force is weak, and graphene accelerates faster under the action of electric field force, resulting in a small response time to electric field. When the interlayer binding energy coefficient *γ*_g_ is 0.26 J/m^2^, 0.23 J/m^2^, and 0.20 J/m^2^, the response time is 8.9 ns, 6.3 ns and 5 ns, respectively [Figure 7(c)]. The increase of friction will reduce the velocity of graphene and increase the response time [Figure 7(b)]. When the friction coefficient *α*_f_ is 0.67*α*_f0_, *α*_f0_, and 1.5*α*_f0_, the response time is 4.3 ns, 6.3 ns, and 9.3 ns, respectively [Figure 7(d)]. This means that the response time and modulation frequency can be further improved by adjusting the interlayer binding energy and friction.

At present, UV modulation is mainly realized by the internal modulation which presents a large noise, causing the rate to decrease rapidly while increasing the output power. In this work, an external modulation with a low noise, whose rate is less affected by the output power of the light source. When superlubrication is introduced, the modulation exhibits a high rate and a wide FWHM, and it can modulate signal sources of different wavelengths at the same time, avoiding effectively the interference among different signal sources. The comparison of modulation strategies in specific UV systems is shown in Table 1. It is worth noting that although our results are obtained through simulation, all parameters used in the simulation are based on experimental results, such as the friction coefficient and the interlayer binding energy. And the geometrical design of the metamaterial substrate is also achievable experimentally. Therefore, the design should provide theoretical guidance for developing a UV modulator based on a graphene micromechanical system.

**Table 1:** Comparison of modulation strategies in UV systems.

Modulation mode	Sources	Modulation rate	Ref.
Internal modulation	Nd:YAG laser	600 Hz	[53]
Internal modulation	Hydrogen-xenon flashlamp	40 KHz	[54]
Internal modulation	Mercury-xenon lamp	400 KHz	[11]
Internal modulation	UV LED	20 KHz	[55]
Internal modulation	UV LED	5 KHz	[12]
Internal modulation	UV LED	1 KHz	[56]
External modulation	No Restrictions on the light source	50 MHz	This work

## 4 Conclusions

In this study, we used the superlubricity of graphene and surface plasmonic metamaterial to realize UV electro-optic modulation of graphene NEMS. By tuning the gap between a suspended graphene and a metamaterials substrate through applied voltage, the strong localized light field near the plasma metamaterials is utilized to realize wide-range regulation of light reflection. The graphene is periodically deflected up and down by periodic voltage drive. The numerical results show that the modulation depth of the electro-optic modulator can be significantly improved by combining the graphene in the UV region to form a surface plasmonic metamaterial structure with a strong local field. The absolute variation in reflection of monolayer graphene can be more than 0.4. The modulated reflection of three graphene layers has an absolute change of reflection of more than 0.6 and a relative change of reflection of 0.98. Additionally, the superlubricity further improves the performance of UV electro-optic modulator based on graphene NEMS due to its extremely low friction coefficient, with a modulation voltage equal to or less than 150 mV and a modulation response speed up to nanoseconds. The findings indicate that the proposed graphene-plasmonic metamaterial devices with mechanical modulation will have important applications in UV communication fields.
